# *In vitro* interactions of nystatin and micafungin combined with chlorhexidine against *Candida albicans* isolates

**DOI:** 10.18502/cmm.8.1.9208

**Published:** 2022-03

**Authors:** Maede Salehi, Ali Malekzadeh Shafaroudi, Maryam Daryani, Alireza Khalilian, Fatemeh Ahangarkani, Tahere Molania

**Affiliations:** 1 Department of Oral Medicine, Faculty of Dentistry, Mazandaran University of Medical Sciences, Sari, Iran; 2 Department of Dental Research Center, Mazandaran University of Medical Sciences, Sari, Iran; 3 Student Research Committee, School of Dentistry, Mazandaran University of Medical Sciences, Sari, Iran; 4 Department of Community Medicine, School of Medicine, Thalassemia Research Center, Mazandaran University of Medical Sciences, Sari, Iran; 5 Antimicrobial Resistance Research Center, Communicable Diseases Institute, Mazandaran University of Medical Sciences, Sari, Iran

**Keywords:** *Candida albicans*, Chlorhexidine, Combination, Fluconazole, Micafungin, Nystatin

## Abstract

**Background and Purpose:**

Oral candidiasis has become a growing problem in hospitals worldwide, and the development of antifungal drug resistance in *Candida* species constitutes a serious concern. This study aimed to evaluate the *in vitro* efficacy of nystatin, and micafungin with chlorhexidine against fluconazole-resistant and fluconazole-sensitive *Candida albicans* (*C. albicans*) isolates.

**Materials and Methods:**

In this experimental-laboratory study, a total of 20 fluconazole-resistant (n=10) and fluconazole-susceptible (n=10) *C. albicans* strains were obtained from the reference culture collection of the Invasive Fungi Research Center in Mazandaran University of Medical Sciences, Sari, Iran. *In vitro* combination of nystatin and micafungin with chlorhexidine was performed using a microdilution checkerboard method based on the Clinical and Laboratory Standards Institute guideline.

**Results:**

Micafungin had the highest antifungal activity against *C. albicans* susceptible and resistant strains, with a Geometric mean of (GM) =0.008µg/ml and GM=0.008µg/ml, followed by nystatin with GM=0.06µg/ml and GM=0.042µg/ml and chlorhexidine with GM=0.25µg/ml and GM=0.165µg/ml against *C. albicans* resistant and sensitive strains, respectively. The interaction of micafungin and nystatin with chlorhexidine showed a synergistic interaction against most *C. albicans* strains. In addition, no antagonistic interaction was observed between micafungin, nystatin, and chlorhexidine against *C. albicans* strains.

**Conclusion:**

The synergistic interaction of micafungin with chlorhexidine against azole-resistant *C. albicans* suggests an alternative approach to overcome antifungal drug resistance. However, further studies are needed for *in vivo* evaluation.

## Introduction

Over the past 20 years, oral candidiasis has been a growing problem in hospitals worldwide due to an increase in predisposing factors, including long-term treatment with broad-spectrum antibiotics, more than 72 h in the ICU, treatment with immunosuppressive drugs, intravenous catheters, and injectable nutrition [ 1 - 3 ]. 

Although *Candida albicans* (*C. albicans*) is still the most common cause of oral candidiasis, the prevalence of non-*albicans*-induced oral candidiasis has increased. The main antifungal drugs used to treat oral candidiasis include azole agents, especially fluconazole, echinocandins (micafungin), and polyenes (nystatin). Unfortunately, resistance to these drugs has recently increased significantly [ 4 , 5 ]. Resistance to fluconazole has been observed among Candida species in various regions worldwide. The emergence of rare antifungal-resistant *Candida* species, such as *C. auris*, *C. kefir*, and *C. lusitania*, has been reported in many centers [ 6 - 12 ].

Treatment of oral candidiasis caused by rare *Candida* species has been controversial due to a lack of knowledge on drug susceptibility profile, intrinsic antifungal resistance, or multiple antifungal-resistant strains [ 13 ]. Due to the limitations of antifungal agents and the antifungal resistance phenomenon, combination therapy can be an effective strategy against the therapeutic challenges of oral candidiasis due to resistant species. In this regard, antifungal medications can be combined with chlorhexidine to disinfect the body before surgery and sterilize surgical instruments [ 14 ]. The antimicrobial activity of chlorhexidine is due to the ability of these agents to destroy the cell wall. Chlorhexidine is also used to clean wounds, delay the formation of dental plaque, and treat oral candidiasis. Body wounds, tooth discoloration, and allergic reactions are listed as chlorhexidine side effects [ 15 ]. 

Recent research on the combined effects of disinfectants on similar cases has led to desirable results based on the potentiating effect of these drugs. However, the combined effects of chlorhexidine with antifungal drugs against resistant *C. albicans* have not yet been studied. Therefore, designing a study to evaluate these combined effects seemed to be necessary. This study aimed to evaluate the *in vitro* effectiveness of antiseptic drugs in combination with antifungal agents against fluconazole-resistant and fluconazole-sensitive *C. alibicans* isolated from oral candidiasis.

## Materials and Methods

### Characterization of isolates

In this study, a panel of 20 *C. albicans* isolates, including fluconazole-resistant (n=10) and fluconazole-sensitive (n=10) isolates, were obtained from the reference culture collection of the Invasive Fungi Research Center (IFRC) at the Mazandaran University of Medical Sciences, Sari, Iran. All tested isolates have been previously identified by sequencing of internal transcribed spacer ribosomal DNA (ITS-rDNA) regions and MALDI-TOF mass spectrometer assay (MALDI Biotyper OC version 3.1, Bruker Daltonics, Bremen, Germany) [ 10 , 11 ]. Isolates were sub-cultured on Sabouraud Dextrose Agar (SDA, Difco) at 30 °C to ensure purity and viability. The study protocol was approved by the Ethics Committee of Mazandaran University of Medical Sciences, Sari, Iran (IR.MAZUMS.REC.1397.2980).

### In vitro antifungal susceptibility testing

Nystatin (Bristol-Myers-Squib, Woerden, Netherlands), Micafungin (Astellas Pharma, Ibaraki, Japan), and Chlorhexidine (PubChem) minimum inhibitory concentrations (MICs) were determined according to the broth microdilution guideline (M60) of Clinical and Laboratory Standards Institute (CLSI). In this study, *Candida albicans* (ATCC 64124) was used as a reference strain.

### In vitro combination testing by the checkerboard method

The interactions of nystatin and micafungin with chlorhexidine against fluconazole-resistant and fluconazole-sensitive *C. albicans* isolates were investigated using a microdilution checkerboard method based on the CLSI reference technique with 96-well microtiter plates [ 16 ]. The prepared drug dilutions were four times the final concentration in terms of volume. The concentration ranges of drugs depended on the MIC results of each isolate. Briefly, 50 µl of each concentration of chlorhexidine was dispensed into the columns of 1 to 10, and 50 µl of nystatin or micafungin was added to the rows of A to G of 96-well microplates. The H row and column 11 contained chlorhexidine and nystatin or micafungin alone, respectively. In addition, column 12 was used as the drug-free growth control. For each drug combination plate, 100 µl of inoculum was added to all the wells. The inoculum was prepared using fresh colonies, and their density was adjusted to 1-3×10^3^ CFU/ml at 530 nm wavelength to a percentage transmission within a range of 75-77%. Plates were incubated at 35°C and examined visually after 24 h to determine the MIC values for the drugs separately and in combination with others.

The MIC endpoints were determined using a reading mirror and were defined as the lowest concentration of drug that significantly reduced growth (less than 50%) compared with the growth of a drug-free control. For the determination of drug interactions, the fractional inhibitory concentration index (FICI) was calculated as FICI = FIC_A_ + FIC_B_ = (C_A_/MIC_A_) + (C_B_/MIC_B_), where MIC_A_ and MIC_B_ are the MICs of drugs A and B alone, and C_A_ and C_B_ are the concentrations of the drugs in combination, in all wells corresponding to an MIC. The interaction was considered synergistic at FICI ≤0.5, indifferent at >0.5 to ≤4.0, and antagonistic at >4 [ 16 ].

## Results

Table 1 summarizes the MIC ranges, MIC_50_, MIC_90_, and geometric means (GM) MIC nystatin and chlorhexidine with micafungin. In terms of GM MIC, micafungin had the highest antifungal activity against all *C. albicans* isolates (GM MIC=0.008 µg/ml), Followed by nystatin with a GM MIC=0.06 µg/ml against fluconazole-resistant *C. albicans* and a GM MIC=0.042 µg/ml against fluconazole-sensitive *C. albicans* isolates, as well as chlorhexidine with a GM MIC=0.25 µg/ml against fluconazole-resistant *C. albicans* and a GM MIC=0.165µg/ml against fluconazole-sensitive *C. albicans* isolates. The highest range of MIC was observed in chlorhexidine against fluconazole-resistant *C. albicans* isolates (0.5-0.031 µg/ml) and fluconazole-sensitive *C. albicans* isolates (0.25-0.063 µg/ml).

**Table 1:** *In vitro* susceptibilities of *C. albicans* isolates to chlorhexidine, nystatin, and micafungin

Strain	Number	Antifungal agent	MIC (mg/L)												MIC rang	MIC_90_	MIC_50_	G mean
			0.001	0.002	0.004	0.008	0.016	0.031	0.063	0.125	0.25	0.5	1	2				
Fluconazole- resistance *C.albican*	N=10	Chlorhexidine						1	4	1	3	1			0.031-0.5	0.063	0.25	0.25
		Nystatin						2	7		1				0.031-0.25	0.063	0.063	0.06
		Micafungin			3	7									0.004-0.008	0.008	0.008	0.008
Fluconazole-susceptible *C.albicans*	N=10	Chlorhexidine							2	2	6				0.063-0.25	0.25	0.25	0.165
		Nystatin					1	5	4						0.016-0.063	0.031	0.063	0.042
		Micafungin		1	2	7									0.002-0.008	0.008	0.008	0.008

MIC: minimum inhibitory concentrations; CHG: chlorhexidine; NST: nystatin; MFG: micafungin; GM: geometric means

Table 2 summarizes the results of the combination of nystatin and micafungin with chlorhexidine. Using the interpretation of FICI, in the combination of nystatin and chlorhexidine from all *C. albicans* isolates, synergistic interactions were shown on 7 (35%) isolates with FICI≤0.5. Moreover, the combination of micafungin and chlorhexidine interactions showed synergistic interactions, as 14 (70%) out of all *C. albicans* isolates had FICI≤ 0.5. However, other isolates have shown indifferent

**Table 2:** Minimum inhibitory concentrations of nystatin and micafungin with chlorhexidine alone and in combination against *C. albicans* isolates

Strains	CHG	CHG+NST		FICI/INT	CHG	CHG+MFG		FICI/INT
		MIC (µg/mL)				MIC (µg/mL)		
		NST	CHG/NST			MFG	CHG/MFG	
IFRC 27	0.125	0.016	0.031 /0.001	0.31 / SYN	0.125	0.008	0.016/0.002	0.37/SYN
IFRC 600	0.25	0.031	0.063 /0.008	0.52/ IND	0.25	0.008	0.031/0.002	0.37 / SYN
IFRC 37	0.063	0.25	0.016/0.063	0.50 / SYN	0.063	0.008	0.016/0.002	0.50/SYN
IFRC 604	0.063	0.031	0.016/0.008	0.51/ IND	0.063	0.008	0.016/0.001	0.37/SYN
IFRC 614	0.25	0.063	0.031 /0.016	0.37 / SYN	0.25	0.004	0.008/0.001	0.28/SYN
IFRC 25	0.125	0.031	0.031/0.008	0.50/ SYN	0.125	0.008	0.031/0.002	0.49/SYN
IFRC 120	0.25	0.063	0.063 /0.016	0.50 / SYN	0.25	0.008	0.016/0.002	0.31/SYN
IFRC 13	0.063	0.031	0.016/0.008	0.51/ IND	0.063	0.008	0.016/0.001	0.37/SYN
IFRC 18	0.125	0.063	0.008/0.031	0.55/ IND	0.125	0.004	0.008/0.001	0.31/SYN
IFRC 15	0.25	0.063	0.063/0.016	0.51 / IND	0.25	0.008	0.016/0.004	0.56/ IND
IFRC 24	0.25	0.063	0.063 /0.031	0.74 / IND	0.25	0.008	0.016/0.001	0.18/SYN
IFRC 14	0.25	0.063	0. 063/0.016	0.53 / IND	0.25	0.008	0.016/0.002	0.31/SYN
IFRC 10	0.063	0.063	0.016/0.008	0.37/ SYN	0.063	0.004	0.008/0.001	0.37 / SYN
IFRC 1055	0.031	0.031	0.008/0.004	0.38/ SYN	0.031	0.008	0.016/0.002	0.76/IND
IFRC 1262	0.25	0.063	0.016 /0.031	0.55 / IND	0.25	0.004	0.008/0.001	0.28/SYN
IFRC 1261	0.25	0.063	0.125/0.125	2.48/ IND	0.25	0.008	0.016/0.004	0.56/ IND
IFRC 38	0.5	0.063	0.125/0.063	1.5/ IND	0.5	0.008	0.008/0.004	0.51/ IND
IFRC 603	0.063	0.031	0.016/0.008	0.51/ IND	0.063	0.002	0.002/0.001	0.53 /IND
IFRC 616	0.25	0.063	0.125/0.031	0.99/ IND	0.25	0.004	0.031/0.001	0.37/SYN
IFRC 1260	0.063	0.031	0.016/0.008	0.51/ IND	0.063	0.008	0.016/0.004	0.75/IND

MIC: minimum inhibitory concentrations; CHG: chlorhexidine; NST: nystatin; MFG: micafungin; FICI: fractional inhibitory concentration index; SYN: synergism; IND: indifference

interaction with 0.5<FICI≤4, and none of the isolates showed antagonist interactions.

## Discussion

The combination of chlorhexidine with micafungin showed a synergistic interaction against most *C. albicans* isolates in the present study. In 2017, Scheibler *et al*. reviewed dental and medical literature concerning the use of nystatin and chlorhexidine in oral medicine and reported that nystatin and chlorhexidine are gold-standard antimicrobial mouthrinses for *Candida* spp. They suggested that further studies should investigate interactions of other drug combinations to improve the therapeutic management of oral candidiasis [ 17 ]. Many *in vitro* studies of antifungal drugs have shown that the drug combination can broaden the spectrum of antifungal treatment, increase the fungicidal effect, reduce the toxicity of drugs, and reduce the antifungal resistance phenomenon. For instance, Monteiro *et al*. reported that silver nanoparticles combined with nystatin and chlorhexidine digluconate demonstrated synergistic antibiofilm activity.

On the other hand, Alvendal *et al*. [ 18 ] reported that in eradicating *C. albicans*, chlorhexidine digluconate eliminated the biofilm more effectively than fluconazole [ 19 ]. According to Garcia-Cuesta *et al*., nystatin and amphotericin B are the most commonly used topical drug for treating oral candidiasis. Oral administration of fluconazole is also known to be very effective in treating this infection [ 20 ]. However, recent studies in the United States, Europe, and Asia have shown increased resistance of *Candida* species to fluconazole and echinocandins [ 5 , 11 ]. Due to the limitations of antifungal agents and the development of antifungal resistance, combination therapy can be an effective strategy for the therapeutic challenges of candidiasis caused by resistant species [ 21 ]. Studies of antifungal drugs with different mechanisms of action against *Candida* species have also been performed. 

On the other hand, many studies have shown that different concentrations of each drug combination can have consequences ranging from antagonism to synergy. Host factors strongly influence the antifungal agent [ 22 ]. Many mechanisms of synergy have been proposed between existing antifungal drugs. For example, terbinafine and azoles disrupt the function of fungal cells through inhibition of biosynthesis. Another mechanism of synergism involves the simultaneous inhibition of different cellular targets, such as synthesizing echinocandins and amphotericin B [ 23 ]. A combination of antifungal drugs can be used for treatment; however, it should be noted that the wrong combination can reduce the effect of fungicides and sometimes increase toxicity. Similar to synergy, the mechanism of antagonism is different. Antagonism may be due to the direct action of two drugs that reduce the availability of each target in the fungal cell [ 24 ]. Most clinical studies conducted on combined antifungal therapy against yeasts have been performed for the treatment of *Cryptococcal* infections. Several studies have reported the use of combined antifungal therapy for the treatment of endocarditis caused by *Candida* species, fungal central nervous system infection, azole-resistant *C. glabrata* infections, Candida pyelonephritis, and *Candida* endophthalmitis [ 25 ]. A randomized clinical trial compared the antifungal effects of fluconazole alone and in combination with amphotericin B and showed that combination therapy with fluconazole and amphotericin B could clear blood infection faster. Echinocandins in combination with azoles are also a known treatment option for invasive candidiasis. The combination of posaconazole with caspofungin and micafungin has been investigated in an animal model [ 26 ]. In another study, Chen et al. showed *in vitro* and *in vivo* synergism effects of posaconazole in combination with caspofungin against echinocandin resistant isolates [ 27 ]. 

A multicenter study against azole- or echinocandin-resistant *C. albicans*, *C. glabrata*, and *C. parapsilosis* also concluded that synergistic effects could be obtained in combination with more antifungal drugs [ 28 ]. Rodriguez *et al*. also investigated the combined effect of micafungin and fluconazole on 105 clinical isolates (including 15 isolates of *C. albicans*, 20 isolates of *C. dubliniensis*, 15 isolates of *C. glabrata*, 20 isolates of *C. krusei*, and 15 isolates of *C. tropicalis*) and reported a synergistic effect on 33%, 26%, and 7% of *C. albicans*, *C. tropicalis*, and *C. glabrata* isolates, respectively [ 29 ]. Due to the emergence of resistant non- *albicans* species with different susceptibility patterns and given the fact that the treatment strategy is based solely on the identification of *Candida* with conventional mycological methods, further investigation is needed for the accurate identification of the species and the application of effective drugs or combination therapy to combat drug resistance. Further clinical trials are required before the generalization and daily use of antifungal drug combinations in treating invasive candidiasis.

## Conclusion

The combination of chlorhexidine with micafungin exhibited synergistic activity against azole-resistant *C. albicans*. This can be used as an alternative approach to overcome antifungal drug resistance. However, further studies are required for *in vivo* evaluation.

## Acknowledgments

This study has been extracted from a dissertation financially supported by Mazandaran University of Medical Sciences (Thesis code: 2980). The researchers would like to thank the authorities in the Student Research Committee of Mazandaran University of Medical Sciences, Sari, Iran, for supporting this project.

## Authors’ contribution

M.S., T.M., M.D., and F.A. collected the data, and A.K. performed the statistical analyses, interpreted the data, drafted, and revised the manuscript for important intellectual content.

T.M. and A.M.S. reviewed the analyses and the final version of the manuscript. M.D. and AMS interpreted the data, revised the manuscript for important intellectual content, and approved the final version. All authors have read and approved the manuscript. 

## Conflicts of interest

All authors declare that they have no conflict of interest regarding the publication of this study.

## Financial disclosure

Not applicable.
